# Food Insecurity Pre- and Post the COVID-19 Pandemic and Economic Crisis in Lebanon: Prevalence and Projections

**DOI:** 10.3390/nu13092976

**Published:** 2021-08-27

**Authors:** Samer Kharroubi, Farah Naja, Marwa Diab-El-Harake, Lamis Jomaa

**Affiliations:** 1 Department of Nutrition and Food Sciences, Faculty of Agricultural and Food Sciences, American University of Beirut, Riad El-Solh, P.O. Box 11-0236, Beirut 1107-2020, Lebanon; sk157@aub.edu.lb (S.K.); md106@aub.edu.lb (M.D.-E.-H.); lj18@aub.edu.lb (L.J.); 2 Department of Clinical Nutrition and Dietetics, Research Institute of Medical & Health Sciences (RIMHS), College of Health Sciences, University of Sharjah, Sharjah 27272, United Arab Emirates; fnaja@sharjah.ac.ae; 3 Faculty of Agricultural and Food Sciences, American University of Beirut, Riad El-Solh, P.O. Box 11-0236, Beirut 1107-2020, Lebanon; 4 Department of Human Sciences, College of Health and Sciences, North Carolina Central University, Durham, NC 27707, USA

**Keywords:** COVID-19, pandemic, economic crisis, food insecurity, trends, projections, modeling, GWP, Lebanon

## Abstract

The coronavirus (COVID-19) pandemic has had serious repercussions on the global economy, work force, and food systems. In Lebanon, the pandemic overlapped with an economic crisis, which threatened to exacerbate food insecurity (FI). The present study aims to evaluate the trends and projections of FI in Lebanon due to overlapping health and economic crises. Data from Gallup World Poll (GWP) 2015–2017 surveys conducted in Lebanon on nationally representative adults (*n* = 3000) were used to assess FI trends and explore its sociodemographic correlates. Predictive models were performed to forecast trends in FI (2018–2022), using GWP data along with income reduction scenarios to estimate the impact of the pandemic and economic crises. Pre crises, trend analyses showed that FI could reach 27% considering wave year and income. Post crises, FI was estimated to reach on average 36% to 39%, considering 50–70% income reduction scenarios among Lebanese population. FI projections are expected to be higher among females compared to males and among older adults compared to younger ones (*p* < 0.05). These alarming findings call for emergency food security policies and evidence-based programs to mitigate the burden of multiple crises on the FI of Lebanese households and promote resilience for future shocks.

## 1. Introduction

The coronavirus (COVID-19) pandemic is an unprecedented crisis on a global scale that has led to a dramatic loss of human lives with serious repercussions extending beyond health [1,2]. The pandemic, along with the associated social distancing and lockdown measures, has had remarkable impacts on the global economy, work force, and food systems that are still being unraveled [1,3]. The protective public health measures that were required globally to suppress the transmission of the virus caused massive economic and social shocks. The global economy has plunged into a recession, and the economic effects of COVID-19 are proving to be far worse than the 2008–2009 financial crisis [4]. In addition, the pandemic severely damaged employment across all sectors due to the social distancing policies, especially the services sector, which declined by 50% globally [5]. Border closures, lockdowns, and disruptions in supply chains, markets, and trade have also put the global food system under immense strain and are affecting food supply and demand, as well as people’s access to sufficient and nutritious sources of food [6].

Since the declaration of the COVID-19 as a global health crisis in March 2020, multiple reports and studies were published exploring the impact of the pandemic on various dimensions of food insecurity (FI), including food availability, access, and utilization [7,8,9]. Restrictions on movement, disruption of food supply chains, and increased food price volatilities threatened the food and nutrition security of population groups worldwide [7,10,11]. Although the pressures that this pandemic posed are universal, the repercussions were not experienced equally across countries and their populations [12]. Low-to-middle-income countries with weak economic infrastructures and countries with prolonged wars and humanitarian crises were particularly exposed to the adverse effects of the pandemic. For example, Arab countries that are home to only 5% of the world’s population [13] continue to host more than 40% of the forcibly displaced populations worldwide [14]. Due to decades of protracted conflicts and wars, many of the Arab countries were expected to be disproportionately affected by COVID-19 [15,16]. In early 2020, approximately 8.3 million people in the Arab region were predicted to fall into poverty due to the pandemic, and the number of food-insecure and undernourished people was projected to increase dramatically by two million individuals [17]. In addition, the long-standing high dependence on food imports within the Arab world, particularly for wheat and protein-rich foods, continues to pose an additional threat to their food security and resilience during the pandemic [17,18].

Lebanon is a small, middle-income country in the Middle East and North Africa (MENA) region with a population of six million individuals and a long history of political and economic instabilities. The country continues to have the highest concentration of refugees per capita worldwide, with more than one-quarter of its population comprising displaced individuals fleeing from war-torn countries in the region, including Syria, Iraq, and Palestine [19]. Prior to the pandemic, Lebanon was facing major economic challenges as the outcome of years of political and sectarian feuds, institutional corruption, and ineffective fiscal policies [20,21,22]. With Lebanon being the world’s third most indebted country and with unemployment rates reaching 30% in 2019, national protests erupted on 17 October 2019 demanding major political, economic, and social reforms [23,24]. Since then, the country has been undergoing a significant economic fallout with income losses, inflation, and devaluation of the Lebanese currency that pushed more than half of the country below the poverty line [25]. Yet, the COVID-19 pandemic further exacerbated a devastating economic crisis and exposed the weaknesses of the social protection systems in the country. Furthermore, the year 2020 was disastrous for Lebanon with the tragic Beirut port blast taking the lives of more than 200 individuals, wounding thousands, and destroying a large part of the capital. The colossal effect of the port blast meant the destruction of the grain silos and the full disruption of the import and export capacity of a country that relies on imports for 80% of its food basket [26,27].

There have been limited studies assessing the prevalence and projection trends of FI in Lebanon and the region using robust data [28,29,30,31] and none, to our knowledge, that explored these trends post pandemic and in light of the pressing geo-political, economic, and social challenges facing its populations. Prior to the COVID-19 pandemic, analyses of FI in the League of Arab States using GWP data showed that countries with political unrest had the highest prevalence of severe FI compared to more politically stable countries [29]. FI was also associated with poor subjective wellbeing among Arab youth, particularly in less stable countries [31]. However, the recent pandemic and its economic repercussions are threatening to further exacerbate FI in countries undergoing political unrest and challenged by other aggravating factors, such as the case of Lebanon. The latter presents a unique setting to explore FI prevalence and trends amidst the pandemic and other overlapping crises and to showcase potential strategies to alleviate FI, particularly among the most vulnerable and affected population groups.

The present study aimed to estimate the impact of overlapping crises that Lebanon has been enduring on FI prevalence. More specifically, the study aimed to (1) explore the trends of FI in Lebanon using Gallup World Poll data (GWP; 2015–2017), (2) evaluate the sociodemographic and economic correlates of FI, and (3) forecast the trends of FI (2018–2022) using the empirical GWP data while considering multiple income reduction scenarios based on recent evidence. Findings from the present study were also used to provide evidence-based and context-specific recommendations to alleviate the deterioration of the food and nutrition security of vulnerable population groups in Lebanon. 

## 2. Materials and Methods

### 2.1. Study Population 

Data for the present study were drawn from Gallup World Poll (GWP) surveys for years 2015–2017, conducted in Lebanon on nationally representative samples of the population aged 15 years and older (*n* = 3000) [20]. Data collection took place over three wave years: between 5 May and 13 June in 2015, between 30 March and 27 April in 2016, and between 20 April and 29 May in 2017. Face-to-face interviews with study participants were held in a household setting and lasted 60 min on average. Households were the primary sampling unit and were selected using a three-stage stratified cluster sampling approach, whereby the strata were the six Lebanese governorates, and the clusters were drawn from the 26 districts (Caza) in Lebanon (stage 1). Clusters within districts were chosen on the basis of probability proportional to size sampling. Within each cluster, households were selected using a systematic sampling approach (stage 2). In the third stage, respondents were randomly selected by the latest birthday or the Kish grid method [32]. Further details about the sampling framework are presented elsewhere [32].

### 2.2. Food Security Measure

Individual-level FI status was measured in the GWP surveys using the Food Insecurity Experience Scale (FIES). The latter is an experience-based measure of FI developed by the Food and Agriculture Organization ‘Voices of the Hungry’ project [33]. FIES consists of an eight-point scale regarding people’s actual experiences in accessing food in the previous 12 months. Respondents were asked whether, at any time during the previous 12 months, they worried about their ability to obtain enough food, their household run out of food, or they were forced to compromise the quality or quantity of the food that they ate owing to limited availability of money or other resources to obtain food. Respondents were assigned a score value of “1” for any specific question that they answered “yes” to and “0” if their answer was “no”. For each respondent, the assigned values for the eight questions were summed to obtain a raw score ranging from 0 to 8. The total score was used to classify individual-level FI status as follows: (1) food-secure, with raw scores = 0; (2) mild FI, with raw scores = 1–3; (3) moderate FI, with raw scores = 4–6; (4) severe FI, with raw scores = 7–8 [33]. In the present study, FI was further recoded into two categories: (1) food-secure (raw score = 0), and (2) food-insecure (raw score ≥ 1); the latter included individuals experiencing mild, moderate, or severe FI).

### 2.3. Sociodemographic and Economic Variables

Data collected as part of the GWP surveys included demographic variables such as age, sex, and marital status. In the present study, age was coded as a categorical variable, with a cutoff point of 10 years: 15–24 years, 25–34 years, 35–44 years, 45–54 years, and 55 years and above. For sex, two categories were considered: “males” and “females”. Marital status was recoded as married (1) and not married (0); the latter included separated, widowed, divorced, single, and never married. Socioeconomic variables examined in the present study included educational level, employment status, and yearly household income. Responses regarding education were coded into three categories: elementary, completed elementary education or less (up to 8 years of basic education); secondary, completed some secondary education up to 3 years (9–15 years of education); tertiary, completed 4 years of education beyond “high school” and/or received a 4 year college degree [32]. Additionally, employment status was recoded into two categories: employed, part-time and full-time work; unemployed, unemployed or out of workforce. Household income per capita was estimated based on monthly income including wages, salaries, remittances from family members, farming, and all other sources [32]. Respondents were categorized into five categories, as per capita income: richest 20%, fourth 20%, middle 20%, second 20%, and poorest 20%. 

### 2.4. Statistical Analysis

Data were analyzed using Stata/MP (version 15, StataCorp., College Station, TX, USA). For the summary of the data, descriptive statistics were used, such as frequencies and proportions. Economic and sociodemographic correlates of FI were explored using simple and multiple logistic regression analyses. In this study, we focused on the dichotomous FI variable: being food-secure versus being food-insecure. More precisely, FI was divided into two categories: (1) “food-secure” (FIES raw score of zero), and (2) “food-insecure” (FIES raw score of one or more). In all analyses, the ‘food-secure’ status was used as the reference group. In the simple logistic regression, each of the economic and sociodemographic variables was used separately as an independent variable, while FI status (food-secure versus food-insecure) was used as a dependent variable. All economic and sociodemographic variables that showed significance in the simple logistic models were included in the final multiple logistic regression models as independent variables. Results from the logistic regression models were expressed as odds ratio (ORs) and adjusted odds ratio (AORs) with 95% confidence intervals (CIs). All reported *p*-values were based on two-sided tests and were compared with a significance level of 5%. 

Projections of FI prevalence and trends (2018–2022) were based on two data sources: (1) FI rates from GWP data (2015–2017), and (2) alternative income reduction scenarios/assumptions (50%, 60%, and 70%). These assumptions were based on the rising poverty and unemployment rates reported in the country between 2019 and 2020 among the Lebanese population post COVID-19, economic crisis, and the most recent Beirut port explosion [2,25,34,35]. Four multiple logistic regression models were used to assess the trends in FI between 2018 and 2022. The dependent variable in these models was FI, whereby FI was a binary variable (food-secure vs. food-insecure). Model 1 was used to predict the trends in FI (2018–2022) using the GWP 2015–2017 empirical data. Models 2–4 were used to predict FI trends using the three different income reduction scenarios presented above whilst considering other sociodemographic correlates of FI. Wave year was further included in each of the four models. 

The predictive probability $p_{i}$ of observing FI for respondent *i* under the four assumed multiple logistic regression models mentioned above is given by the following mathematical formula:

(1) $$p_{i}=\frac{\exp\left(\beta_{0}+\beta_{1}X_{1i}+\ldots +\beta_{k}X_{ki}\right)}{1+\exp\left(\beta_{0}+\beta_{1}X_{1i}+\ldots +\beta_{k}X_{ki}\right)},$$

where *i* = 1, 2, …, *n* represents respondents, $X_{ki}$ indicates values for the *k* covariates for respondent *i* (i.e., economic and sociodemographic variables), and $\beta_{k}$ indicates the regression parameters estimated within the logistic model. 

## 3. Results

Table 1 displays the sociodemographic and economic characteristics of the total study population, classified by FI, using GWP data (2015–2017). On average, FI was experienced by 13% of the study population over the three wave years. Pooled analysis showed that there was an equal gender distribution in the study sample (50.8% females), and more than half were married (55.6%). Slightly less than one-quarter of the pooled study sample (22.2%) had an elementary education level or lower with the remaining having either secondary education (26.2%) or tertiary education and above (21.5%). Approximately 60% of the sample was employed, and household income distribution was presented in quintiles (see Table 1). 

Simple and multiple logistic regression analyses showed significant associations between sociodemographic and economic characteristics of the study population and FI, as presented in Table 1. Odds of FI were found to be higher with age, yet lower among participants with higher educational level and household monthly income, even after adjusting for other significant correlates (all *p*-trend ≤ 0.001). For example, results from the adjusted model showed that participants who completed secondary or tertiary education levels were significantly less likely to experience FI compared to those with elementary education or less (OR = 0.73; 95% CI: 0.57, 0.95 and OR = 0.55; 95% CI: 0.37, 0.81, respectively). In addition, the likelihood of experiencing FI significantly decreased across income quintiles with participants in the highest income quintile (richest 20%) having significantly lower odds of FI compared to lowest income group (poorest 20%) (OR = 0.18; 95% CI: 0.12, 0.28, *p*-trend < 0.001). Females were 67% more likely to experience FI as compared to males, even after adjusting for FI correlates (OR = 1.67; 95% CI: 1.30, 2.16). Participants aged 45–54 years and those aged 54 years and above were also twice as likely to experience FI compared to younger participants (15–24 years) (OR = 2.05; 95% CI: 1.40, 3.04; OR = 2.28; 95% CI: 1.54, 3.38, respectively, *p*-trend < 0.001).

**Figure 1:**
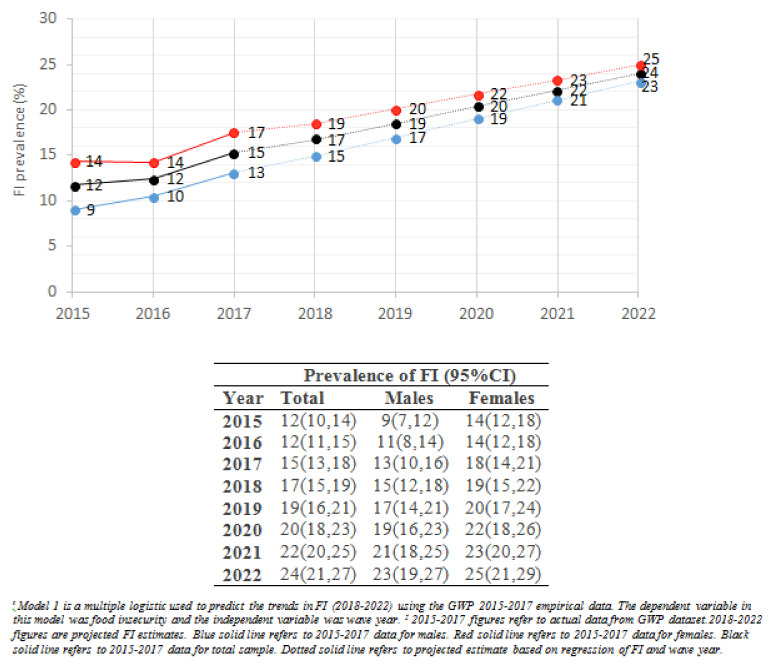
Trends and projections of FI (2018–2022) among Lebanese adult population using **model 1**^ŧ^ (including wave year) from GWP data (2015–2017) ^‡^.

Figure 1 shows the prevalence of FI across the 3 years (2015–2017) and the predicted FI rates for years 2018–2022 using model 1, with wave year as an independent variable. Results showed a steady increase in the proportions of study participants who would be food insecure over the years. According to model 1, FI rates were projected to reach, on average, 20% and 24% of all survey participants by 2020 and 2022, respectively. Projections of FI were also noted to be higher for females compared to males, reaching 25% and 24% by 2022, respectively (see Figure 1).

**Figure 2:**
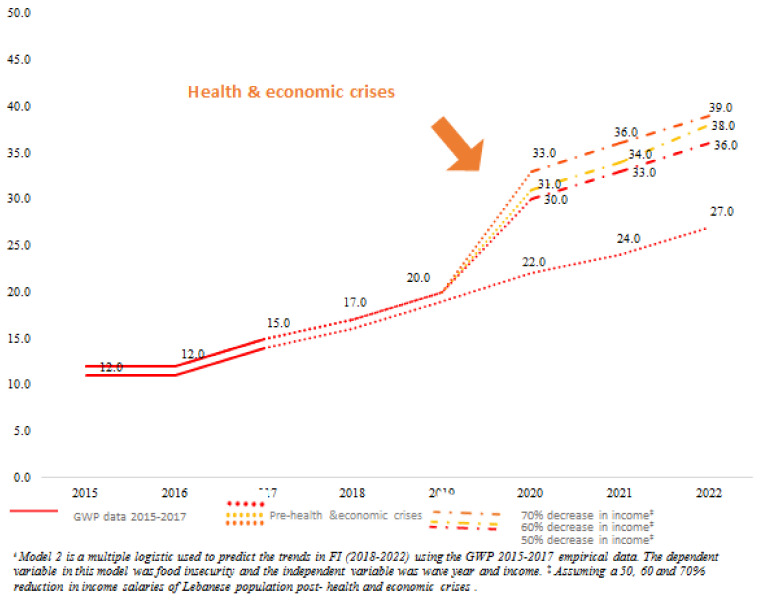
Projections of FI (FI) prevalence among Lebanese adult population using **model 2**^ŧ^ (including year and income) and based on alternative income reduction scenarios.

Figure 2 presents the FI projections for years 2018–2022 calculated from *model 2.* In brief, this model takes into consideration wave year and income from the GWP data (2015–2017), along with three alternative income reduction scenarios (50%, 60%, and 70%). Projections using GWP wave year (2015–2017) and income data only (i.e., using pre-crises data in Lebanon) showed that, on average, FI prevalence might reach 27% by 2022. However, using the GWP wave year (2015–2017) and the three scenarios for income reduction (i.e., reflecting changes post the economic and health crises in the country), projections for FI are expected to range between 33% and 36% in 2021 and between 36% and 39% by 2022 (Figure 2). 

**Figure 3:**
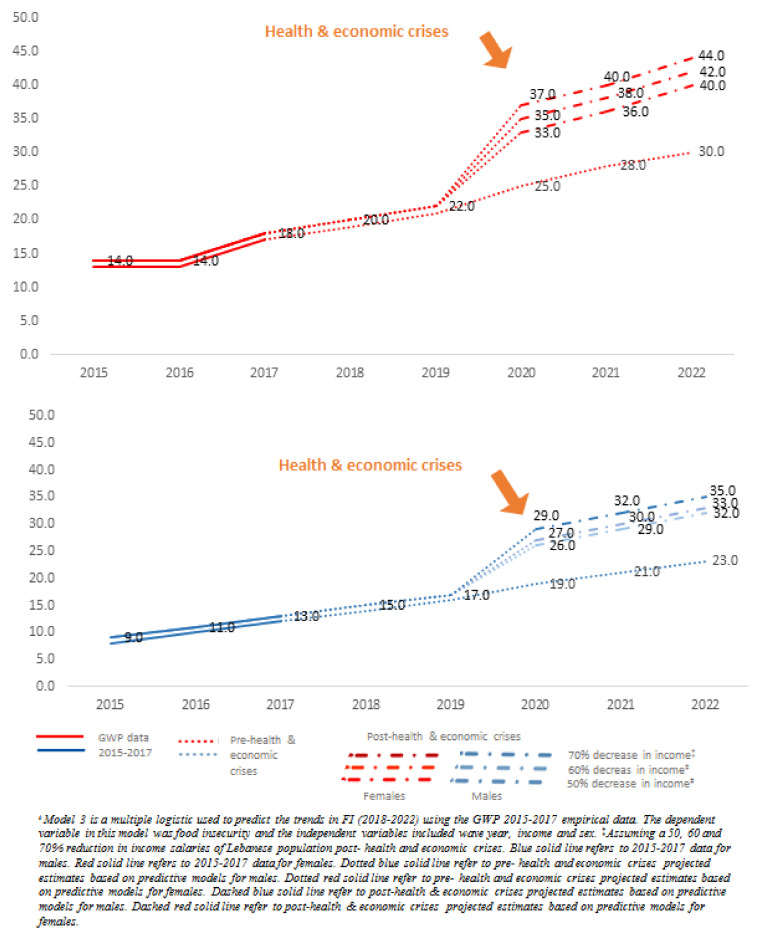
Projections of FI (FI) prevalence among Lebanese adults by sex, using **model 3^ŧ^** (including year, income, and sex) and based on alternative income reduction scenarios.

Figure 3 presents the FI projections for years 2018–2022 using *model 3,* considering wave year, income, and sex from the GWP data (2015–2017), along with the three alternative income reduction scenarios. Higher trends of FI were estimated for females compared to males across the years. Projections in FI prevalence ranged between 40% and 44% for females and between 32% and 35% for males by 2022, considering the three income reduction scenarios (50%, 60%, and 70%).

**Figure 4:**
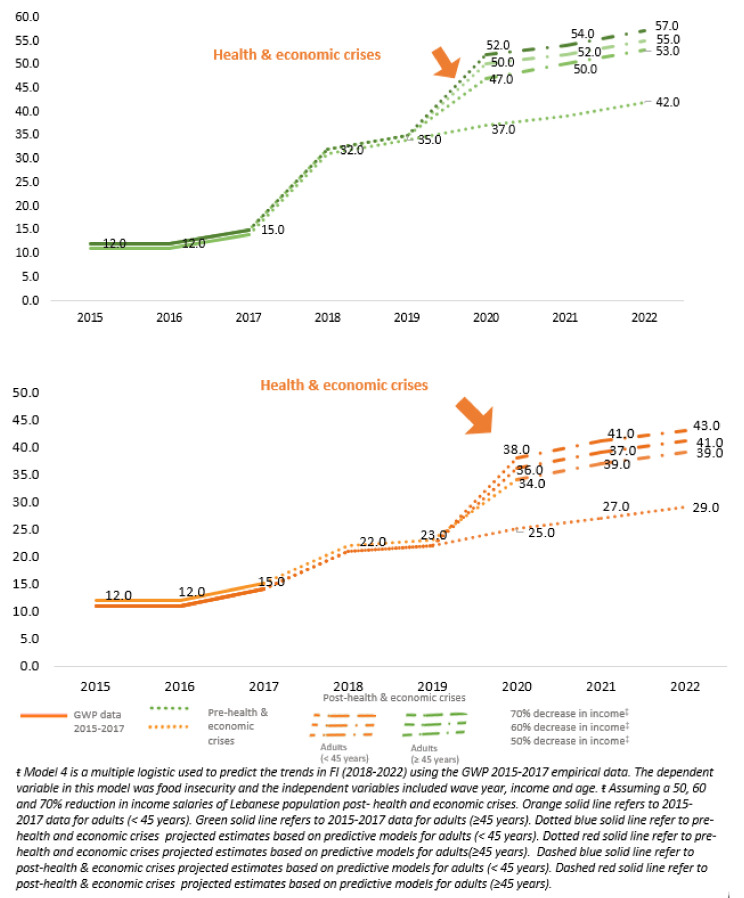
Projections of FI (FI) prevalence among Lebanese adult population by sex and age, using **model 4^ŧ^** (including year, income, and age) and based on alternative income reduction scenarios.

Figure 4 shows the FI projections for years (2018–2022) using *model 4,* which takes into consideration wave year, income, and age from the GWP data (2015–2017) and the three alternative income reduction scenarios (50%, 60%, and 70%). Projections in FI prevalence for older age groups (≥45 years) were estimated to be higher than those for younger age groups (15–45 years old). More specifically, results showed that FI among adults ≥45 years was projected to reach 53%, 55%, and 57%, by 2022, considering the three income reduction scenarios, respectively. On the other hand, the prevalence of FI amongst those <45 years was expected to range between 39% and 43% by 2022. 

## 4. Discussion

To our knowledge, this study is the first to evaluate the trends of FI in Lebanon and to estimate the impact of multiple crises the country has been undergoing on FI projections using empirical data and scenario-based modeling techniques. Our study findings showed that FI was experienced, on average, by 13.1% of the population in Lebanon for the period of 2015–2017. Using the GWP empirical data, our predictive models also showed that, prior to the health and economic crises, FI was projected to reach up to 20% to 24% of the population by 2020 and 2022, respectively. Yet, post the COVID-19 pandemic and the economic crisis, and considering the GWP data and income reduction scenarios applied in the present study, FI in Lebanon is predicted to reach alarming rates. 

Our results are worth comparing to previous data published from Lebanon while considering recent developments at global and local levels with the COVID-9 pandemic, the economic recession, and their implications on food security status. A previous review on FI in the region using the GWP 2016 data showed that Lebanon had, at the time, the lowest levels of moderate to severe FI (5.2%) compared to high-income countries, as well as other middle-income countries in the MENA region, such as Jordan (27.3%) and Egypt (29.8%). In fact, Lebanon was classified along with other Gulf countries in terms of its food security levels despite its much lower per capita GDP [36]. The relatively higher food security status of Lebanon at the time was partly attributed to the favorable climate, water, and soil conditions in the country that increased the diversity of food production compared to other countries in the region with semiarid to arid conditions [37,38,39]. In addition, Lebanon’s economy was reliant mostly on tourism, foreign investments, and remittances [40], which injected money into the country, potentially shielding its population from high levels of FI. However, over the years, Lebanon has been slowly witnessing more water shortages and a series of droughts and other extreme weather events affecting its agricultural productivity and food security [41]. In parallel, geopolitical changes, political instability, and poor fiscal policies, along with an accumulated debt since the end of the Lebanese civil war, have all led to a cascade of events leading to the eruption of national protests in 2019 and a spiraling economic crisis. These political, economic, and environmental pressures were threatening the food security status of the country even prior to the pandemic. With the COVID-19 pandemic and related confinement measures, nearly one-third of the Lebanese population was pushed further into unemployment, and one-fifth of the population witnessed a reduction in their salaries [35]. In addition, half of the Lebanese population reportedly felt worried they would not have enough food to eat during the early stages of COVID-19 spread, and UN agencies projected an increase in the number of Lebanese and displaced population groups requiring basic assistance to maintain their lives and livelihoods [35].

Additionally, Lebanon remains a net food importer at a deficit of 2.1 billion USD, as per the 2019 figures, with nearly 80% of cereal consumption being met through imports. Following the financial crisis and the pandemic, the alarming public debt, wide fiscal deficit, and contracted GDP growth forced the country to decrease both general and food imports. In the first half of 2020, there was an annual decrease of 41.6% in the unloaded total imports weight at the port of Beirut and a 14.6% decrease in the unloaded imports weight of food and beverages. Forecasts also confirmed the persistence in trade deficits at −10.1 billion USD in 2020 and −9.6 billion USD in 2021 [26]. With the tragic Beirut port blast that took place on 4 August 2020 and the destruction of the main artery for the country to import and store food, among other vital resources, the country’s food security status was at a pivotal junction [26]. The destruction of the port of Beirut negatively affected supply chains and the timely availability of grains to the whole country, as well as further exacerbated the food security status of the country. 

Our predictive models showed that pre crises, FI could reach 27% considering wave year and income. However, post the compounding crises witnessed in Lebanon since 2019, FI would be estimated to reach on average 36% to 39%, considering 50–70% income reduction scenarios among the Lebanese population. In fact, recent developments and reports highlight the worsening of the financial situation of the country in line with our worst-case scenario projections. According to the World Bank Lebanon Economic Monitor (LEM), Lebanon is enduring a severe and prolonged economic depression likely to rank in the top three most severe crises globally since the mid-19th century [42]. The World Bank warned that the monetary and financial conditions remain highly volatile in the country, within the context of a multiple exchange rate system. In addition, the effect on prices has resulted in surging inflation rates, reaching 88.2% in 2021, and this is expected to further increase with the ongoing political gridlock and financial free fall that Lebanon is undergoing. More than half the population is expected to be below the national poverty line, with the bulk of the labor force paid in local devaluated currency and suffering from plummeting purchasing power. With the unemployment rate on the rise, an increasing share of households is facing difficulty in accessing food and basic services, including healthcare [42]. These trends are alarming and point in the direction of other countries, including Venezuela, Sudan, and Yemen, that have witnessed similar political and economic crises over the past two decades and are currently listed among the countries with the highest inflation rates globally [43], which, in 2020, were among the most affected countries by the pandemic facing acute food insecurity crises [44].

Results from the present study also showed that females and older age respondents were significantly more likely to be food-insecure compared to males and younger respondents. FI projections, using the pre-crises GWP data and income reduction scenarios, showed that FI could range between 32% and 35% among males and between 40% and 44% among females by 2022. In addition, our models projected FI prevalence to reach up to 57% among middle-aged and older Lebanese adults (≥45 years) using the worst-case scenarios of a 70% reduction in income. The obtained results regarding the association of FI with sociodemographic correlates (age, income, and gender) were in concordance with the scientific literature. A recent in-depth analysis of gender differences in FI conducted by Broussard et al. (2018) using the GWP data showed that females continue to have a higher probability of experiencing FI in many regions of the world. Lower educational attainment, unemployment, and restricted household incomes among women relative to men contribute the most to the gender gap in FI globally and regionally [36,45]. In the MENA region, gender differences in income accounted for over 70% of the observed gender gap in FI, and a lower education among women relative to men also contributed significantly to the observed differences (4–45%) [45]. Previous studies conducted in Lebanon [28,46] and other similar middle-income countries [30,36,45,47,48] also showed that low educational attainment, unemployment, and poor income were associated with higher odds of FI. 

### 4.1. Recommendations and Potential Strategies for Alleviation of Food Insecurity in Lebanon

The projected trends in FI using the different models and assumptions are rather alarming. These findings, along with the recent developments that have been taking place in the country in the past 2 years, call for immediate actions to adopt evidence-based and context-specific policies and strategies to address the various dimensions and pillars of food security, including food availability, access, and utilization. 

In terms of food availability, Lebanon is heavily dependent on food imports with 85% of the country’s food basket, including staple foods such as cereals and sugars, being imported from other countries to meet consumer demands [34,49]. Despite the country’s favorable climate conditions compared to the region, the agricultural production in the country has been largely limited due to massive urbanization post the civil war, as well as low public investment in this sector (general budget allocation is only around 0.5% of the entire state budget) [49]. With the onset of COVID-19 and the looming economic crisis, an emergency agriculture plan was released in April 2020 by the Lebanese Ministry of Agriculture recommending an increase in local agricultural production, particularly of high-value “cash crops”, including fresh fruits and vegetables, as well as a reduction in the gap between food supply and food demand. In addition, the plan proposed that the government provide in-kind aid and small loans to local farmers, import staple foods directly from producing countries, and increase domestic cereal production [50]. Although this emergency plan was considered a step in the right direction, it still lacked a clear coordinated strategy with other relevant ministries and sectors to ensure the feasibility and sustainability of such short- to medium-term projects and to ensure that the food produced would be healthy, affordable and accessible to consumers. Instead, a comprehensive national food security strategy is still lacking to mitigate the impact of the pandemic on the various components of our local food system from reducing disruptions in the food supply chains, to promoting local food production and resilience, and stabilizing the economy to promote food and nutrition security.

With respect to food access, Lebanon has been witnessing in the past year and a half a spiral devaluation of the Lebanese currency against the US dollar and a spike in food prices that has affected the purchasing power of all consumers and their dietary choices [51,52]. These effects were most pronounced amongst the most vulnerable households and those dependent on food assistance for survival. Recent estimates show that more than 237,000 individual from the poorest and most vulnerable Lebanese households are dependent on food assistance through the National Poverty Targeting Program [53], and 358,000 of Syrian refugees receive cash assistance from the joint UNHCR/WFP programs [54]. The high food and nonfood price inflation combined with the loss of jobs and salary cuts aggravated by the pandemic-related lockdown measures are amongst the aggravating factors that reduced the households’ abilities to afford adequate and sufficient food. These developments, together with our projected trends, call for immediate measures to mitigate FI through monitoring and stabilizing the market food prices, subsidizing essential food commodities, and expanding the existing social welfare and food assistance programs to provide essential assistance in the short term while also supporting livelihoods in the long-term [49,55,56]. 

It is also worth noting that shifts in purchasing and consumption behaviors (food utilization) were also reported in the country with the onset of the pandemic. As concomitantly witnessed in several countries in the MENA region and globally [57,58], consumers in Lebanon adopted increased panic-buying and food-hoarding behaviors. In addition, consumers increasingly purchased processed foods that are cheaper and have longer shelf lives compared to the more nutritious, perishable food items [35]. The impact of the health pandemic was further compounded by the economic crisis leading to inflation, spikes in food prices, and shortage of many food supplies and goods. These changes may have contributed to the panic-buying and hoarding behaviors that were noted among the population. Thus, food and cash transfer programs, as well as other social protection programs that exist in the country, need to work further with retailers, local farmers, and food producers to ensure fresh food supply chains remain open and provide affordable healthy and nutritious foods for consumers [58,59]. In parallel, public health and community-based interventions can be mobilized to increase nutrition education and awareness to assist households in preparing and consuming healthy and nutritious food on limited budgets.

### 4.2. Strengths and Limitations

The present study is the first, to our knowledge, to explore the impact of the overlapping health and economic crises on FI prevalence and trends using mathematical modeling techniques. Other strengths of the study include the rigorous methodology adopted by the GWP together with the national representativeness of the survey and the large sample size. Nevertheless, there are several limitations of the analysis that need to be considered. Due to the cross-sectional nature of the GWP surveys, causality cannot be determined from the study findings. In addition, the present study included a subset of variables provided by the GWP dataset, and future research is needed to further explore the individual, social, and political factors that may affect FI at national and subnational levels. An additional concern is around the limited number of years (2015–2017) used to evaluate and predict the trends in FI prevalence in Lebanon, as well as to forecast the impact of the COVID-19 pandemic and economic crises using these trends. Further studies, with a larger number of years and robust data during and post the pandemic, are required to generate better predictions for future FI rates in the country. 

## 5. Conclusions

The current and projected trends in FI prevalence in Lebanon are alarming and require immediate attention. FI projections are also expected to differentially affect those most vulnerable, including women, older adults, and individuals from lower socioeconomic backgrounds. These alarming findings call for emergency food security policies to mitigate the burden of the health pandemic, along with other overlapping economic and political crises, on the food security of Lebanese households and to promote resilience for future shocks. Evidence-based and context-specific policies and strategies that can address the various dimensions and pillars of food security, including food availability, access, utilization, and stability, are needed. Today, more than ever, food security should be a top national priority alongside health and financial security to ensure that the most vulnerable and marginalized communities are not pushed further into poverty and despair. 

## Figures and Tables

**Table 1:** Associations between the sociodemographic and economic characteristics of the study population and FI in Lebanon using GWP data (2015–2017) ^†^.

		Food Security Status			Logistic Regression	
	Total Sample *n* (%)	Food Secure *n* (%)	Food Insecure *n* (%)	*p*-Value	Unadjusted OR	Adjusted OR ^‡^
**Year**				*0.041*		
2015	1000 (33.30)	883 (88.30)	117 (11.70)		1.0	1.0
2016	1000 (33.30)	876 (87.60)	124 (12.40)		1.07 (0.82,1.40)	1.05 (0.80,1.40)
2017	1000 (33.30)	847 (84.70)	153 (15.30)		1.36 (1.05,1.76)	1.33 (1.02,1.75)
*p-trend*					0.0172	0.1119
**Female**	1525 (50.83)	1291 (49.54)	234 (59.39)	*<0.001*	1.49 (1.20,1.85)	1.67 (1.30,2.16)
**Married**	1668 (55.64)	1444 (55.43)	224 (57.00)	*0.560*	1.07 (0.86,1.32)	-
**Age (years)**				*<0.001*		
15–24 years	597 (19.92)	547 (20.99)	50 (12.69)		1.0	1.0
25–34 years	668 (22.27)	586 (22.49)	82 (20.81)		1.53 (1.06,2.22)	1.64 (1.10,2.42)
35–44 years	633 (21.10)	553 (21.22)	80 (20.30)		1.58 (1.09,2.30)	1.36 (0.92,2.01)
45–54 years	502 (16.73)	418 (16.04)	84 (21.32)		2.20 (1.51,3.19)	2.05 (1.40,3.04)
>54 years	600 (20.00)	502 (19.26)	98 (24.87)		2.13 (1.49,3.06)	2.28 (1.54,3.38)
*p-trend*					<0.001	<0.001
**Educational Level**				*<0.001*		
Elementary or less	667 (22.24)	531 (20.38)	136 (34.52)		1.0	1.0
Secondary	1687 (26.25)	1479 (56.78)	208 (52.79)		0.55 (0.43,0.70)	0.73 (0.57,0.95)
Tertiary (university or higher)	645 (21.51)	595 (22.84)	50 (12.69)		0.33 (0.23,0.46)	0.55 (0.37,0.81)
*p-trend*					<0.001	0.001
**Employment**				*0.043*		
Unemployed	1223 (40.77)	1044 (40.06)	179 (45.43)		1.0	1.0
Employed	1777 (59.23)	1562 (59.94)	215 (54.57)		0.80 (0.65,0.99)	1.19 (0.92,1.54)
**Income (International Dollars)**				*0.001*		
Poorest 20%	498 (16.60)	371 (14.24)	127 (32.23)		1.0	1.0
Second 20%	533 (17.77)	442 (16.96)	91 (23.10)		0.60 (0.44,0.81)	0.65 (0.47,0.88)
Middle 20%	573 (19.10)	501 (19.22)	72 (18.27)		0.42 (0.30,0.58)	0.42 (0.30,0.59)
Fourth 20%	654 (21.80)	592 (22.72)	62 (15.74)		0.31 (0.22,0.43)	0.33 (0.23,0.46)
Richest 20%	742 (24.73)	700 (26.86)	42 (10.66)		0.17 (0.12,0.25)	0.18 (0.12,0.28)
*p-trend*					<0.001	<0.001

^†^ ORs of the dependent variable (food-insecure vs. food-secure) are presented with 95% CIs using simple logistic regression. The food-insecure category included mildly, moderately, and severely food-insecure participants. **^‡^** Adjusted ORs are presented with 95% CIs using multiple logistic regression analysis. The models were adjusted for economic and sociodemographic characteristics found to be significant correlates of FI (year, age, sex, educational level, employment, and income).

## Data Availability

Permission/license to access the GWP dataset was provided to the corresponding author by the Food and Agriculture Organization (FAO) Voices of the Hungry Project. Restrictions apply to the availability of these data, as per the FAO policies.
